# Drug-induced anaphylaxis during general anesthesia in 14 tertiary hospitals in Japan: a retrospective, multicenter, observational study

**DOI:** 10.1007/s00540-020-02886-5

**Published:** 2021-01-09

**Authors:** Tatsuo Horiuchi, Tomonori Takazawa, Masaki Orihara, Shinya Sakamoto, Kazuhiro Nagumo, Shigeru Saito

**Affiliations:** 1 Department of Anesthesiology, Gunma University Graduate School of Medicine, Maebashi, Gunma 371-8511 Japan; 2 Intensive Care Unit, Gunma University Hospital, 3-39-15 Showa-machi, Maebashi, Gunma 371-8511 Japan

**Keywords:** Anaphylaxis, Sugammadex, Rocuronium, Cefazolin, Antibiotics

## Abstract

Since perioperative anaphylaxis occurs suddenly, and it can be life-threatening, anesthesiologists need to have sufficient knowledge of the epidemiology of perioperative anaphylaxis and appropriate coping strategies to deal with it. Recent studies conducted in Western countries reported the characteristics of perioperative anaphylaxis in each country. However, there are few studies of perioperative anaphylaxis in Japan. To bridge the gap between Japan and other countries, the data of 46 anaphylaxis patients at Gunma University Hospital and 13 neighboring hospitals between 2012 and 2018 were collected and analyzed. The recently developed clinical scoring system was combined with a skin test to include only cases with a definite diagnosis. The most common causative agents were sugammadex, followed by rocuronium, cefazolin, and antibiotics other than cefazolin. Furthermore, the characteristics of anaphylaxis for each causative drug were identified. Time from drug administration to appearance of the first symptom was the longest in the cefazolin group. The incidence of canceled operation was the highest in the rocuronium group. Although it is unclear whether the results of this study can apply to Japan as a whole, the information about the agents responsible for perioperative anaphylaxis and the characteristics of anaphylaxis due to each agent would be helpful to anesthesiologists.

**Supplementary Information:**

The online version contains supplementary material available at 10.1007/s00540-020-02886-5.

## Introduction

Intraoperative complications can be minimized by proper monitoring and medication based on relevant preoperative assessment. However, due to the difficulty of predicting the occurrence of anaphylaxis, the risk of developing perioperative anaphylaxis cannot be reduced even with these efforts. Although the severity of reactions ranges from mild to severe, in extreme cases, anaphylaxis may be fatal despite prompt recognition, prolonged adequate resuscitation, and treatment [1]. Therefore, anesthesiologists need to have sufficient knowledge of the epidemiology of perioperative anaphylaxis and appropriate coping strategies to deal with it.

Given these circumstances, large-scale epidemiological studies have recently been conducted in Western countries, and they suggested large differences in the characteristics of perioperative anaphylaxis among countries [2–7]. Unlike the situation in other countries, however, little research has been done in Japan. The last epidemiological survey of perioperative anaphylaxis in Japan was conducted 28 years ago [8]. To bridge the gap between Japan and other countries, we decided to collect and analyze the data of anaphylaxis occurring at Gunma University Hospital and 13 neighboring hospitals in the past 7 years. Since perioperative anaphylaxis is often difficult to diagnose, the recently developed clinical scoring system [9] was combined with a skin test to include only cases with a definite diagnosis. We expect that this research would enable us to recognize the characteristics of perioperative anaphylaxis occurring in Japan.

## Materials and methods

This retrospective, observational study conformed to the standards of the Declaration of Helsinki and was approved by the ethics committee of Gunma University [identification (ID): 150034]. The study was registered with the University Hospital Medical Information Network Clinical Trials Registry (ID: 000022365). Some of the cases included in this study have already been presented in our previous reports, which had different perspectives from the present study [10–14].

The patients diagnosed with anaphylaxis between Jan 2012 and Dec 2018 in Gunma University Hospital and 13 Japanese tertiary hospitals were included in this study. Anaphylaxis was diagnosed only when the following two criteria were fulfilled: (1) evaluation using the clinical monitoring scoring system suggested the possibility of an immediate hypersensitivity reaction (net total score on the clinical grading scale ≥ 8) [9]; and (2) skin tests showed a positive reaction to any of the agents that the patient was exposed to during anesthesia. Patient information regarding the clinical background, the timing of symptom appearance, time from administration of the drug to appearance of the first symptom, types of clinical symptoms, net total score on the clinical grading scale, severity grade, and outcome was collected. The clinical symptoms were classified into four categories, including cardiovascular, respiratory, cutaneous, and gastrointestinal. The severity of clinical symptoms was assessed using the ring and Messmer scale [15].

Continuous variables are reported by the median and interquartile range values. The differences in continuous variables among agents were analyzed with one-way ANOVA or Kruskal–Wallis one-way ANOVA on ranks. Categorical variables were analyzed with Fisher’s exact test. Sigma plot 14.0 (Systat Software Inc, San Jose, CA) or R ver. 3.3.3 was used for the analyses. Differences were considered significant at a *p* value < 0.05.

## Results

A total of 49 patients with suspected anaphylaxis due to their clinical symptoms were initially investigated. Three of these patients had negative skin test reactions for all drugs to which they had been exposed during anesthesia; therefore, 46 patients were ultimately included in this study (Table 1). The common causative agents were as follows: sugammadex (*n* = 13, 28.3%), rocuronium (*n* = 10, 21.7%), cefazolin (*n* = 8, 17.4%), and antibiotics other than cefazolin (*n* = 7, 15.2%) (Table 2, Supplemental Fig. 1).

**Table 1:** Clinical backgrounds, causative agents, timing and onset of the appearance of symptoms, anaphylactic symptoms, severity grades, clinical scores, and outcomes in patients with perioperative anaphylaxis

Patient no.	Age (years)	Sex	Causative agent	Timing	Onset (min)	Symptoms	Severity grade	Clinical score	Cancelled operation	Delayed extubation
1	75	F	Sugammadex	End	3	Ca, Cu	3	25	−	+
2	34	M	Sugammadex	End	1	Ca, Cu	2	19	−	−
3	13	M	Sugammadex	End	5	Ca, Cu, Re	3	30	−	+
4	65	M	Sugammadex	End	< 1	Ca, Cu, Re	3	27	−	+
5	39	M	Sugammadex	End	8	Ca, Cu	2	17	−	−
6	62	M	Sugammadex	End	3	Ca, Ga	3	13	−	−
7	64	F	Sugammadex	End	3	Ca, Cu	2	21	−	+
8	33	F	Sugammadex	End	5	Ca, Cu	2	19	−	−
9	46	F	Sugammadex	End	3	Ca, Cu, Re	3	30	−	+
10	66	M	Sugammadex	End	3	Ca, Cu, Re	3	28	−	−
11	19	F	Sugammadex	End	3	Ca, Cu, Re	3	30	−	+
12	42	F	Sugammadex	End	3	Ca, Cu, Ga	3	15	−	−
13	55	M	Sugammadex	End	3	Ca, Cu	3	17	−	−
14	41	F	Rocuronium	Induction	< 1	Ca, Re	4	35	+	−
15	52	F	Rocuronium	Induction	5	Ca, Cu, Re	3	28	−	−
16	56	F	Rocuronium	Induction	5	Ca, Re	2	22	−	+
17	74	F	Rocuronium	Maintenance	20	Ca, Cu	3	11	−	−
18	16	M	Rocuronium	Induction	5	Ca, Cu	3	23	+	−
19	72	M	Rocuronium	Induction	3	Ca	3	17	+	−
20	38	F	Rocuronium	Induction	3	Ca, Cu, Re	2	22	+	+
21	83	F	Rocuronium	Induction	10	Ca, Cu	2	17	+	−
22	78	F	Rocuronium	Induction	10	Ca, Cu, Re	2	19	−	−
23	36	M	Rocuronium	Induction	3	Ca, Cu, Re	2	28	+	−
24	71	F	Cefazolin	Maintenance	10	Ca, Cu	3	21	+	+
25	26	F	Cefazolin	Maintenance	2	Ca, Re	3	26	+	+
26	67	F	Cefazolin	Maintenance	3	Ca, Cu	2	21	−	−
27	7	F	Cefazolin	Maintenance	10	Ca, Re	3	22	+	−
28	33	F	Cefazolin	Maintenance	20	Ca, Cu	3	12	−	−
29	77	M	Cefazolin	Maintenance	10	Ca, Cu, Re	3	20	−	−
30	51	M	Cefazolin	Maintenance	10	Ca, Cu	3	19	+	−
31	14	M	Cefazolin	Maintenance	10	Ca, Cu	2	24	−	−
32	56	M	Cefoperazone/Sulbactam	Maintenance	5	Ca, Re	4	32	−	+
33	49	F	Cefmetazole	Maintenance	5	Ca, Cu	3	23	−	−
34	36	F	Cefmetazole	Maintenance	5	Ca, Cu	2	17	−	−
35	77	M	Vancomycin	Maintenance	5	Ca, Cu	4	28	−	−
36	86	M	Ceftriaxone	Maintenance	5	Ca, Cu	3	23	+	−
37	44	F	Cefotiam	Maintenance	10	Ca, Cu	3	19	+	−
38	25	M	Fosfomycin	Maintenance	5	Ca, Cu, Re	3	18	+	−
39	54	M	Lidocaine	Induction	< 1	Ca, Cu	3	12	−	−
40	42	F	Mepivacaine	Induction	10	Ca, Cu	3	13	−	−
41	21	M	Flurbiprofen	End	10	Ca, Cu	2	20	−	−
42	61	F	Propofol	Induction	< 1	Cu, Re	2	17	−	−
43	66	M	Fibrin sealant	Maintenance	5	Ca, Cu	3	13	−	−
44	49	F	Fibrin sealant	Maintenance	10	Ca, Cu	2	9	−	−
45	80	F	Atropine	Induction	3	Ca, Cu	2	13	−	−
46	50	M	Iopamidol	Maintenance	10	Ca	3	9	−	−

The timing of symptom appearance was classified into three categories, including induction, maintenance, and end of anesthesia. The induction of anesthesia refers to within 10 min after the start of anesthesia. The end of anesthesia means from the end of surgery to the end of anesthesia. Maintenance of anesthesia is the period between induction of anesthesia and the end of anesthesia. The onset indicates time from drug administration to the appearance of the first symptom. The severity of clinical symptoms was assessed by the Ring and Messmer scale [15]. All patients had a clinical score of 8 or above, suggesting possible anaphylaxis [9]. Delayed extubation was defined as when the patient was extubated after leaving the operating room, or when it took more than two hours from the end of surgery to extubation even in the operating room

*M* male, *F* female, *Ca* cardiovascular signs, *Cu* cutaneous signs, *Re* respiratory signs, *Ga* gastrointestinal signs

**Table 2:** Characteristics of perioperative anaphylaxis classified by causative agent

	Sugammadex	Rocuronium	Cefazolin	Antibiotics	Miscellaneous	All
Number of patients (%)	13 (28.3)	10 (21.7)	8 (17.4)	7 (15.2)	8 (17.4)	46 (100.0)
Background						
Female (%)	6 (46.2)	7 (70.0)	5 (62.5)	3 (42.9)	4 (50.0)	25 (54.3)
Male (%)	7 (53.8)	3 (30.0)	3 (37.5)	4 (57.1)	4 (50.0)	21 (45.7)
Age (years)	47.2 (19.2)	54.6 (22.0)	43.3 (27.0)	53.3 (21.8)	52.9 (17.5)	50.0 (20.9)
Timing						
Induction (%)	0 (0.0)	9 (90.0)^a^	0 (0.0)	0 (0.0)	4 (50.0)	13 (28.3)
Maintenance (%)	0 (0.0)	1 (10.0)	8 (100.0)^b^	7 (100.0)^c^	3 (37.5)	19 (41.3)
End (%)	13 (100.0)^d^	0 (0.0)	0 (0.0)	0 (0.0)	1 (12.5)	14 (30.4)
Onset (min)	3.0 (1.0)	5.0 (7.0)	10.0 (5.3)^e^	5.0 (0.0)	7.5 (8.5)	5.0 (7.0)
Symptom						
Cardiovascular (%)	13 (100.0)	10 (100.0)	8 (100.0)	7 (100.0)	7 (87.5)	45 (97.8)
Respiratory (%)	5 (38.5)	6 (60.0)	3 (37.5)	2 (28.6)	1 (12.5)	17 (37.0)
Cutaneous (%)	12 (92.3)	7 (70.0)	6 (75.0)	6 (85.7)	7 (87.5)	38 (82.6)
Gastrointestinal (%)	2 (15.4)	0 (0.0)	0 (0.0)	0 (0.0)	0 (0.0)	2 (4.3)
Clinical score	22.4 (6.2)	22.2 (6.8)	20.6 (4.1)	22.9 (5.5)	13.3 (3.7)^f^	20.5 (6.4)
Severity grade	3.0 (1.0)	2.5 (1.0)	3.0 (0.8)	3.0 (1.0)	2.5 (1.0)	3.0 (1.0)
Outcome						
Cancelled operation (%)	0 (0.0)	6 (60.0)^g^	4 (50.0)	3 (42.9)	0 (0.0)	13 (28.3)
Delayed extubation (%)	6 (46.2)	2 (20.0)	2 (25.0)	1 (14.3)	0 (0.0)	11 (23.9)

“Antibiotics” refers to antibiotics other than cefazolin. Categorical variables are shown as the actual numbers, and percentages are shown in parentheses. Since age and clinical score were normally distributed, they are shown as means, and standard deviations are shown in square brackets. For onset and severity grade, the median values and interquartile ranges are shown. The timing of symptom appearance was classified into three categories, including induction, maintenance, and end of anesthesia. The induction of anesthesia refers to within 10 min after the start of anesthesia. The end of anesthesia means from the end of surgery to the end of anesthesia. Maintenance of anesthesia is the period between induction of anesthesia and the end of anesthesia. The onset indicates the time from drug administration to the appearance of the first sign. The accuracy of anaphylaxis diagnosis was assessed using the clinical grading scale [9]. The severity of clinical signs was assessed by the Ring and Messmer scale [15]. Delayed extubation was defined as when the patient was extubated after leaving the operating room, or when it took more than two hours from the end of surgery to extubation even in the operating room. The symbols indicate significant differences between groups, and *p* values were 0.005 or less unless otherwise specified

^a^Rocuronium vs. sugammadex, cefazolin, and antibiotics; ^b^Cefazolin vs. rocuronium and sugammadex

^c^Antibiotics vs. rocuronium and sugammadex

^d^Sugammadex vs. all other groups

^e^Cefazolin vs. sugammadex (*p* < 0.05)

^f^Miscellaneous vs. sugammadex, rocuronium, cefazolin, and antibiotics (*p* < 0.05)

^g^Rocuronium vs. sugammadex (*p* < 0.05)

To investigate the characteristics of anaphylaxis for each causative agent, patients were divided into the following five groups: sugammadex, rocuronium, cefazolin, antibiotics other than cefazolin, and miscellaneous. Although there were no significant differences in sex, age, clinical symptoms, and severity grade, the characteristics of anaphylaxis for each causative drug emerged (Table 2). First, the timing of symptom appearance had distinct characteristics. That is, rocuronium-induced anaphylaxis occurred during induction of anesthesia, antibiotic-induced anaphylaxis occurred during maintenance of anesthesia, and sugammadex-induced anaphylaxis occurred at the end of anesthesia. There was only one exception. Second, the time from drug administration to the appearance of the first symptom was the longest in the cefazolin group, significantly longer than in the sugammadex group (*p* < 0.05, one-way ANOVA with the post hoc Dunn’s test). Third, the incidence of cancelled operation was the highest in the rocuronium group, significantly higher than in the sugammadex group (*p* < 0.05, Fisher’s exact test with the post hoc Bonferroni test).

## Discussion

This study summarized the causative agents in 46 cases of perioperative anaphylaxis that occurred in the past 7 years and the characteristics of each causative agent.

Sugammadex was the most common causative agent of perioperative anaphylaxis, which differed from past studies conducted in foreign countries and may be a feature of anaphylaxis occurring in Japanese hospitals. Although various factors are considered, high usage of sugammadex in Japan might be one of the reasons [13, 16]. This situation appears to be different from other countries. For example, the amount of sugammadex used per case of general anesthesia in Japan is expected to be 22.8 times greater than that in the UK [13].

Regarding the neuromuscular blocking agents (NMBAs), rocuronium is also the top causative drug in most countries other than Japan. For example, in Australia, rocuronium was responsible for 56% of cases of NMBA-induced anaphylaxis, succinylcholine 21%, and vecuronium 11% [17]. In the present study, however, no anaphylaxis was observed due to NMBAs other than rocuronium, which might again reflect the high use of rocuronium in Japan [13].

Even greater differences between Japan and other countries may be seen for antibiotic-induced anaphylaxis. Although there are few data on perioperative antibiotics used in each country, NAP6 data from a study conducted in the UK are available: gentamicin was used most often (34.5%), followed by amoxicillin/clavulanic acid (29.8%) and cefuroxime (23.7%) [2]. Although there are no national data on perioperative antibiotic use in Japan, a survey of perioperative drugs we recently conducted at four tertiary hospitals showed that cefazolin was used in 69% of general anesthesia cases, followed by cefmetazole in 12% (unpublished data).

Taken together, the reason for the differences in the causative agents of perioperative anaphylaxis in the current study compared to previous studies might be partially explained by the differences in the drugs used. The fact that sugammadex, rocuronium, and cefazolin account for 67% of causative agents (31 of 46 cases) in perioperative anaphylaxis in the present study might be a prominent feature of perioperative anaphylaxis in Japan.

Although the results of the present study suggest that the timing of anaphylaxis development is clearly different by drug (Table 1), this does not necessarily mean that the causative drug can be determined by the timing alone. For example, drugs other than rocuronium, including lidocaine and propofol, were also included in the “Induction of anesthesia” category (Table 1). An informed guess, which is based on the relationship between the timing of substance exposure and that of symptom appearance, is not a reliable way of determining the cause of a supposed allergic reaction [18]. We would emphasize that the cause of anaphylaxis should be identified by allergy tests such as skin tests. Otherwise, many patients would be at unnecessary risk.

The median time of onset was the latest in the cefazolin group (Table 2). In some cases with a delayed onset, the patient’s body might have already been covered with a surgical drape when the signs of anaphylaxis appeared.

In rocuronium-induced anaphylaxis cases, surgery was cancelled in 60%, the highest rate among the groups. In general, skin testing is recommended to both find a causative agent and identify alternative NMBAs, especially in cases where re-operation is required [16]. Since in patients with anaphylaxis due to rocuronium, skin tests were reported to be positive in 44% for succinylcholine, 40% for vecuronium, and 5% for cisatracurium, cisatracurium is recommended for use as an alternative NMBA [17]. In countries such as Japan where cisatracurium is not available, anesthesia without NMBAs should be considered [16, 19].

This study has several limitations. First, since the location of the participating hospitals was limited to the Kanto region, it is unclear whether the results of this study can be applied to Japan as a whole. Second, since the drugs used at each hospital during the study period were not investigated, the incidence of anaphylaxis for each drug is unknown. Future studies, including the ongoing national epidemiologic study, the Japanese Epidemiological Study for Perioperative Anaphylaxis (JESPA), will address these issues.

In conclusion, the information obtained from the present study regarding the agents responsible for perioperative anaphylaxis and the characteristics of anaphylaxis due to each agent would be useful to anesthesiologists.

## Supplementary Information

Below is the link to the electronic supplementary material.

Supplementary file1 (JPG 799 KB)
