# Supplementary figures and images for: Drug-induced anaphylaxis during general anesthesia in 14 tertiary hospitals in Japan: a retrospective, multicenter, observational study
Source: J Anesth. 2021 Jan 9;35(1):154–60. doi: 10.1007/s00540-020-02886-5 (PMC7840621; doi:10.1007/s00540-020-02886-5)

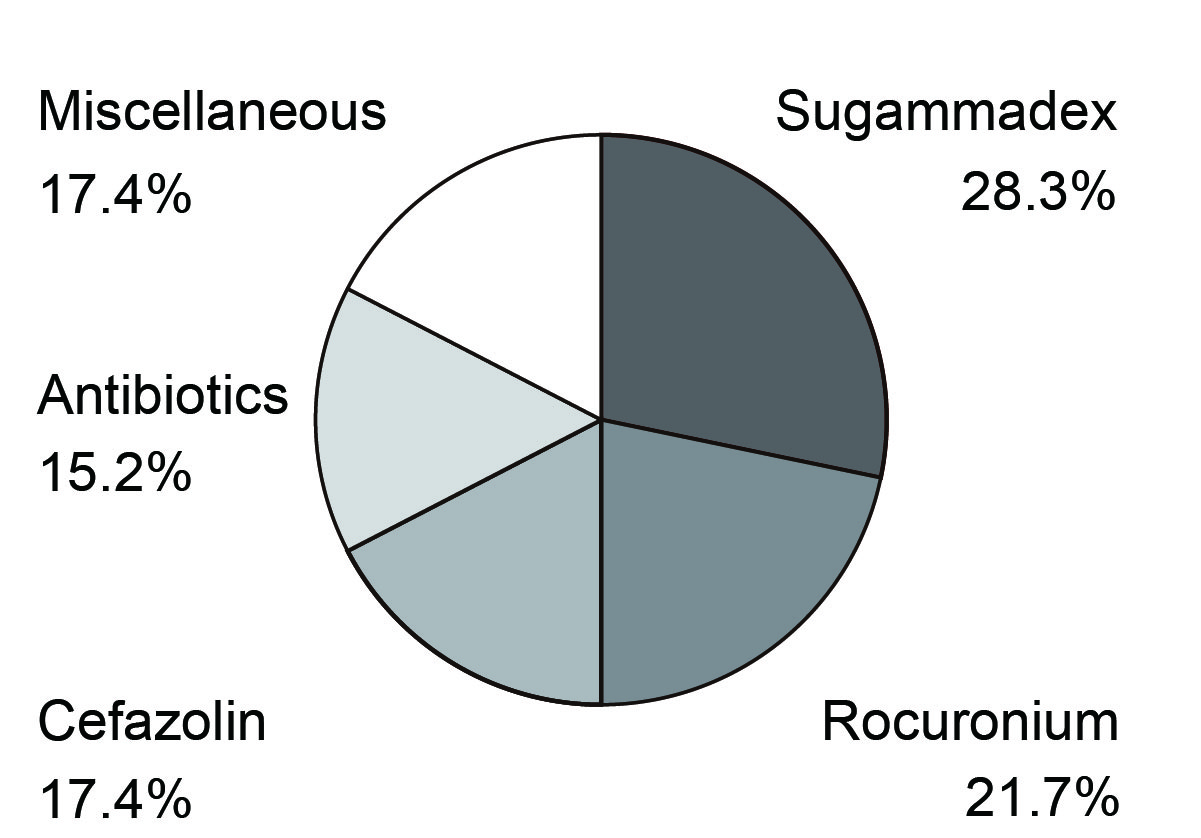

Supplement: Supplementary file 1 — Supplementary file1 (JPG 799 KB) [file 540_2020_2886_MOESM1_ESM.jpg]
